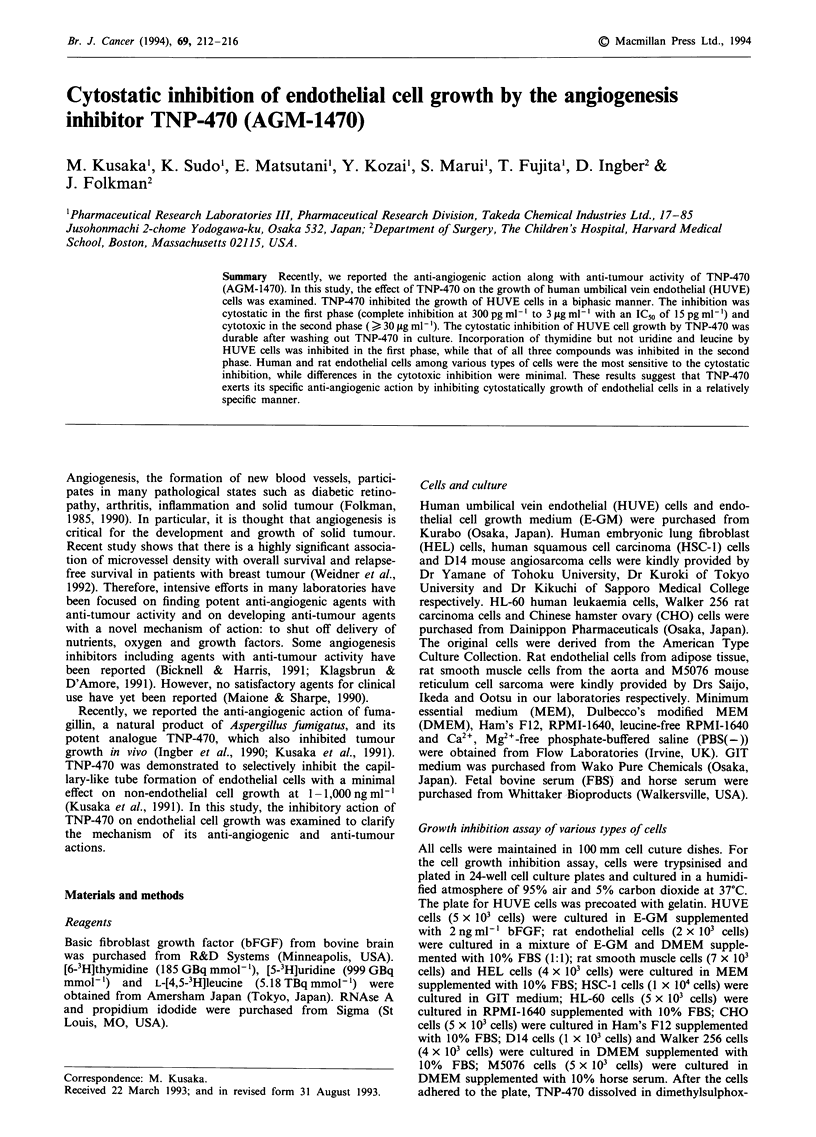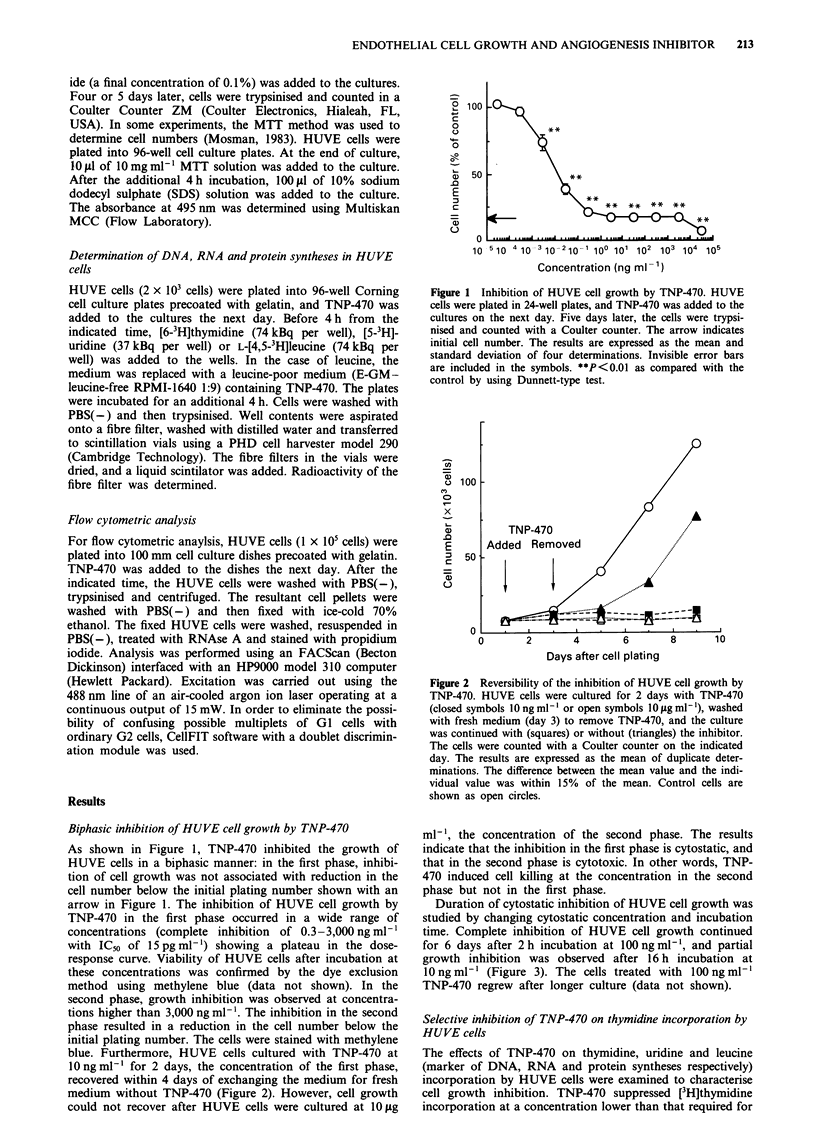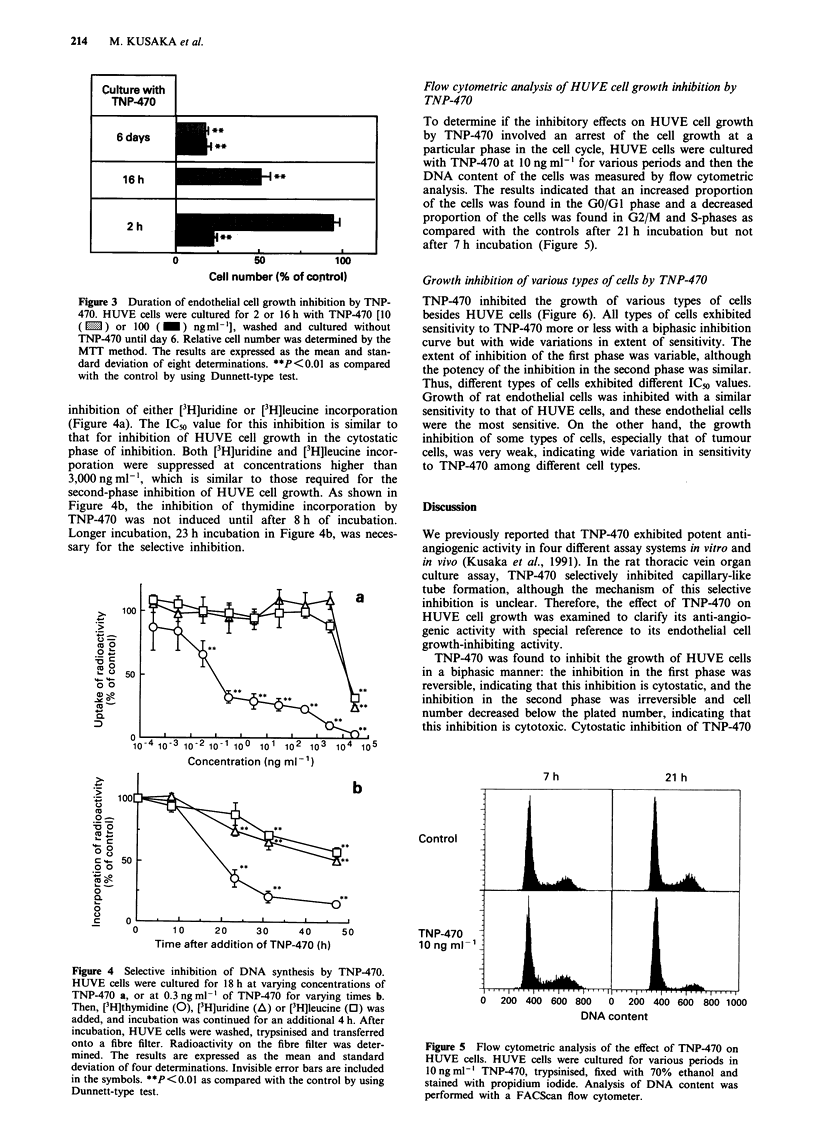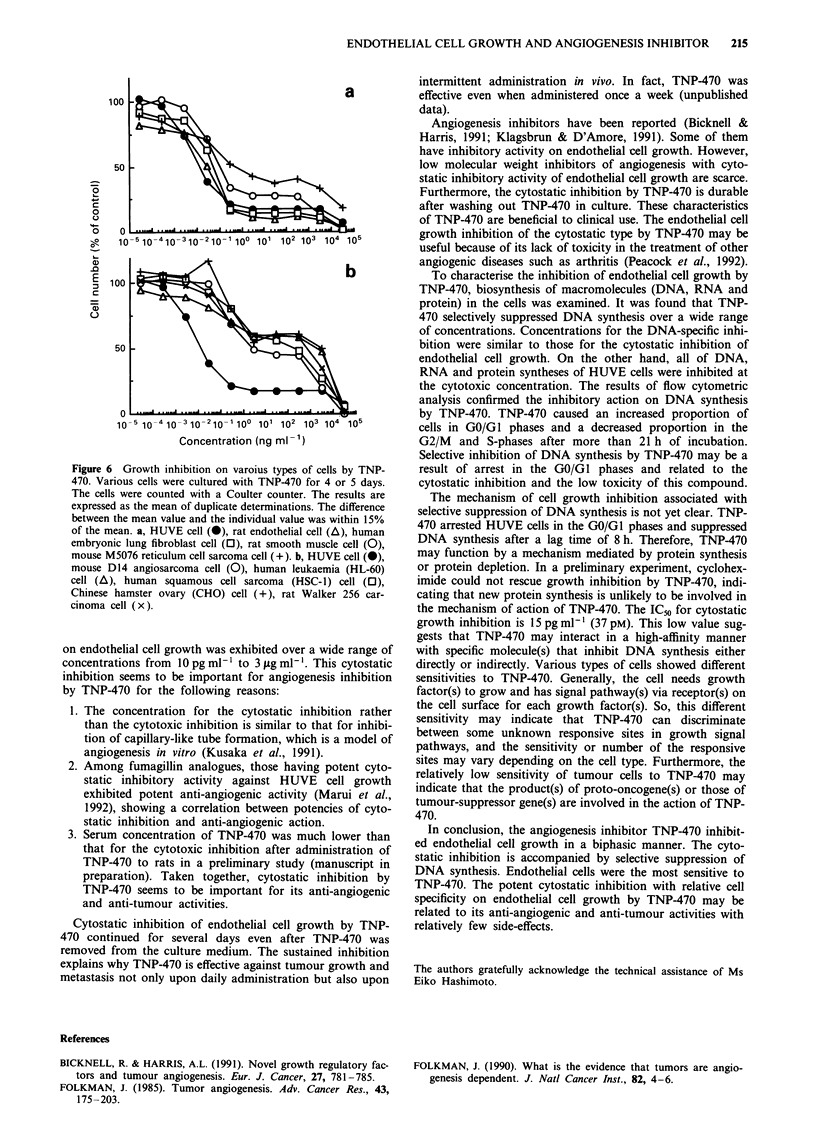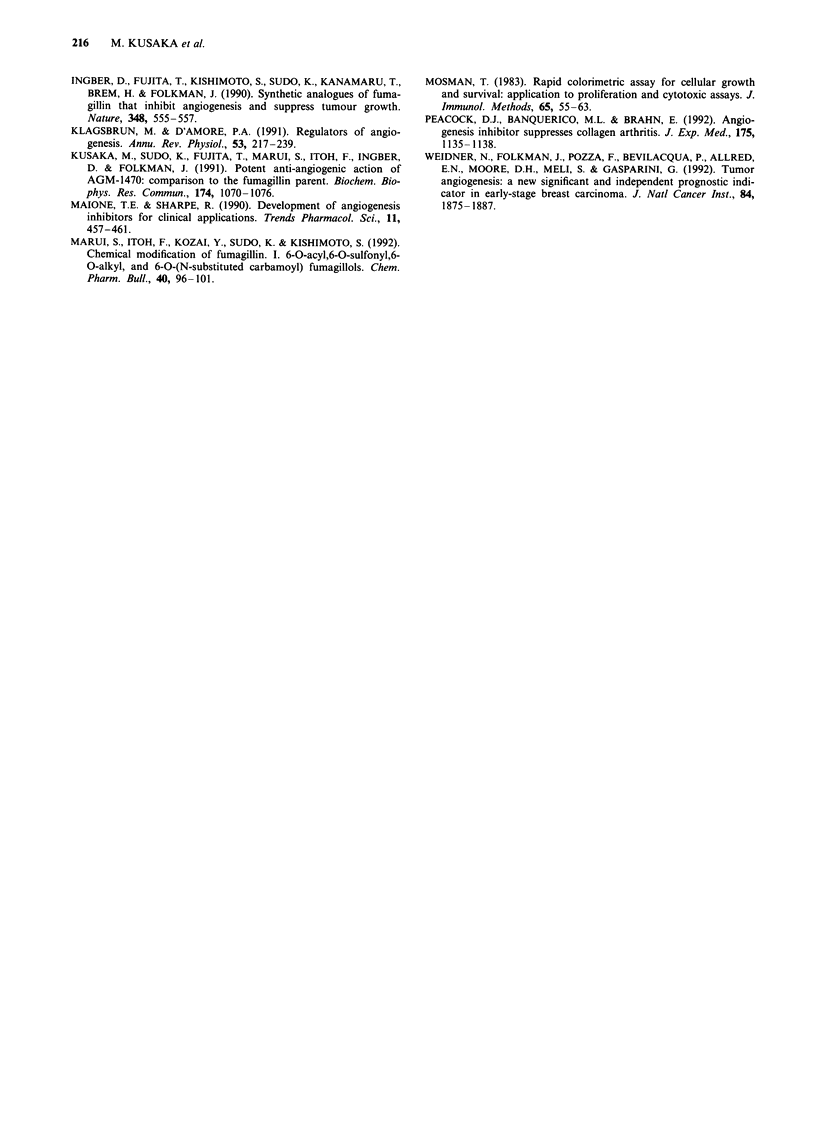# Cytostatic inhibition of endothelial cell growth by the angiogenesis inhibitor TNP-470 (AGM-1470).

**DOI:** 10.1038/bjc.1994.41

**Published:** 1994-02

**Authors:** M. Kusaka, K. Sudo, E. Matsutani, Y. Kozai, S. Marui, T. Fujita, D. Ingber, J. Folkman

**Affiliations:** Pharmaceutical Research Laboratories III, Takeda Chemical Industries Ltd, Osaka, Japan.

## Abstract

Recently, we reported the anti-angiogenic action along with anti-tumour activity of TNP-470 (AGM-1470). In this study, the effect of TNP-470 on the growth of human umbilical vein endothelial (HUVE) cells was examined. TNP-470 inhibited the growth of HUVE cells in a biphasic manner. The inhibition was cytostatic in the first phase (complete inhibition at 300 pg ml-1 to 3 micrograms ml-1 with an IC50 of 15 pg ml-1) and cytotoxic in the second phase (> or = 30 micrograms ml-1). The cytostatic inhibition of HUVE cell growth by TNP-470 was durable after washing out TNP-470 in culture. Incorporation of thymidine but not uridine and leucine by HUVE cells was inhibited in the first phase, while that of all three compounds was inhibited in the second phase. Human and rat endothelial cells among various types of cells were the most sensitive to the cytostatic inhibition, while differences in the cytotoxic inhibition were minimal. These results suggest that TNP-470 exerts its specific anti-angiogenic action by inhibiting cytostatically growth of endothelial cells in a relatively specific manner.


Br. J. Cancer (1994), 69, 212-216 © Macmillan Press Ltd., 1994

### Cytostatic inhibition of endothelial cell growth by the angiogenesis inhibitor TNP-470 (AGM-1470)

M. Kusaka[1], K. Sudo[1], E. Matsutani[1], Y. Kozai[1], S. Marui[1], T. Fujita[1], D. Ingber[2] & J. Folkman[2]

[1]*Pharmaceutical Research Laboratories III, Pharmaceutical Research Division, Takeda Chemical Industries Ltd., 17-85 Jusohonmachi 2-chome Yodogawa-ku, Osaka 532, Japan;* [2]*Department of Surgery, The Children's Hospital, Harvard Medical School, Boston, Massachusetts 02115, USA.*

**Summary** Recently, we reported the anti-angiogenic action along with anti-tumour activity of TNP-470 (AGM-1470). In this study, the effect of TNP-470 on the growth of human umbilical vein endothelial (HUVE) cells was examined. TNP-470 inhibited the growth of HUVE cells in a biphasic manner. The inhibition was cytostatic in the first phase (complete inhibition at 300 pg ml$^{-1}$ to 3 μg ml$^{-1}$ with an $IC_{50}$ of 15 pg ml$^{-1}$) and cytotoxic in the second phase ($\geqslant$30 μg ml$^{-1}$). The cytostatic inhibition of HUVE cell growth by TNP-470 was durable after washing out TNP-470 in culture. Incorporation of thymidine but not uridine and leucine by HUVE cells was inhibited in the first phase, while that of all three compounds was inhibited in the second phase. Human and rat endothelial cells among various types of cells were the most sensitive to the cytostatic inhibition, while differences in the cytotoxic inhibition were minimal. These results suggest that TNP-470 exerts its specific anti-angiogenic action by inhibiting cytostatically growth of endothelial cells in a relatively specific manner.

Angiogenesis, the formation of new blood vessels, participates in many pathological states such as diabetic retinopathy, arthritis, inflammation and solid tumour (Folkman, 1985, 1990). In particular, it is thought that angiogenesis is critical for the development and growth of solid tumour. Recent study shows that there is a highly significant association of microvessel density with overall survival and relapse-free survival in patients with breast tumour (Weidner *et al.*, 1992). Therefore, intensive efforts in many laboratories have been focused on finding potent anti-angiogenic agents with anti-tumour activity and on developing anti-tumour agents with a novel mechanism of action: to shut off delivery of nutrients, oxygen and growth factors. Some angiogenesis inhibitors including agents with anti-tumour activity have been reported (Bicknell & Harris, 1991; Klagsbrun & D'Amore, 1991). However, no satisfactory agents for clinical use have yet been reported (Maione & Sharpe, 1990).

Recently, we reported the anti-angiogenic action of fumagillin, a natural product of *Aspergillus fumigatus*, and its potent analogue TNP-470, which also inhibited tumour growth *in vivo* (Ingber *et al.*, 1990; Kusaka *et al.*, 1991). TNP-470 was demonstrated to selectively inhibit the capillary-like tube formation of endothelial cells with a minimal effect on non-endothelial cell growth at 1-1,000 ng ml$^{-1}$ (Kusaka *et al.*, 1991). In this study, the inhibitory action of TNP-470 on endothelial cell growth was examined to clarify the mechanism of its anti-angiogenic and anti-tumour actions.

### Materials and methods

### *Reagents*

Basic fibroblast growth factor (bFGF) from bovine brain was purchased from R&D Systems (Minneapolis, USA). [6-$^3$H]thymidine (185 GBq mmol$^{-1}$), [5-$^3$H]uridine (999 GBq mmol$^{-1}$) and L-[4,5-$^3$H]leucine (5.18 TBq mmol$^{-1}$) were obtained from Amersham Japan (Tokyo, Japan). RNAse A and propidium idodide were purchased from Sigma (St Louis, MO, USA).

Correspondence: M. Kusaka.
Received 22 March 1993; and in revised form 31 August 1993.

### *Cells and culture*

Human umbilical vein endothelial (HUVE) cells and endothelial cell growth medium (E-GM) were purchased from Kurabo (Osaka, Japan). Human embryonic lung fibroblast (HEL) cells, human squamous cell carcinoma (HSC-1) cells and D14 mouse angiosarcoma cells were kindly provided by Dr Yamane of Tohoku University, Dr Kuroki of Tokyo University and Dr Kikuchi of Sapporo Medical College respectively. HL-60 human leukaemia cells, Walker 256 rat carcinoma cells and Chinese hamster ovary (CHO) cells were purchased from Dainippon Pharmaceuticals (Osaka, Japan). The original cells were derived from the American Type Culture Collection. Rat endothelial cells from adipose tissue, rat smooth muscle cells from the aorta and M5076 mouse reticulum cell sarcoma were kindly provided by Drs Saijo, Ikeda and Ootsu in our laboratories respectively. Minimum essential medium (MEM), Dulbecco's modified MEM (DMEM), Ham's F12, RPMI-1640, leucine-free RPMI-1640 and $Ca^{2+}$, $Mg^{2+}$-free phosphate-buffered saline (PBS(−)) were obtained from Flow Laboratories (Irvine, UK). GIT medium was purchased from Wako Pure Chemicals (Osaka, Japan). Fetal bovine serum (FBS) and horse serum were purchased from Whittaker Bioproducts (Walkersville, USA).

### *Growth inhibition assay of various types of cells*

All cells were maintained in 100 mm cell cuture dishes. For the cell growth inhibition assay, cells were trypsinised and plated in 24-well cell culture plates and cultured in a humidified atmosphere of 95% air and 5% carbon dioxide at 37°C. The plate for HUVE cells was precoated with gelatin. HUVE cells (5 × 10$^3$ cells) were cultured in E-GM supplemented with 2 ng ml$^{-1}$ bFGF; rat endothelial cells (2 × 10$^3$ cells) were cultured in a mixture of E-GM and DMEM supplemented with 10% FBS (1:1); rat smooth muscle cells (7 × 10$^3$ cells) and HEL cells (4 × 10$^3$ cells) were cultured in MEM supplemented with 10% FBS; HSC-1 cells (1 × 10$^4$ cells) were cultured in GIT medium; HL-60 cells (5 × 10$^3$ cells) were cultured in RPMI-1640 supplemented with 10% FBS; CHO cells (5 × 10$^3$ cells) were cultured in Ham's F12 supplemented with 10% FBS; D14 cells (1 × 10$^3$ cells) and Walker 256 cells (4 × 10$^3$ cells) were cultured in DMEM supplemented with 10% FBS; M5076 cells (5 × 10$^3$ cells) were cultured in DMEM supplemented with 10% horse serum. After the cells adhered to the plate, TNP-470 dissolved in dimethylsulphoxide (a final concentration of 0.1%) was added to the cultures. Four or 5 days later, cells were trypsinised and counted in a Coulter Counter ZM (Coulter Electronics, Hialeah, FL, USA). In some experiments, the MTT method was used to determine cell numbers (Mosman, 1983). HUVE cells were plated into 96-well cell culture plates. At the end of culture, 10 µl of 10 mg ml$^{-1}$ MTT solution was added to the culture. After the additional 4 h incubation, 100 µl of 10% sodium dodecyl sulphate (SDS) solution was added to the culture. The absorbance at 495 nm was determined using Multiskan MCC (Flow Laboratory).

*Determination of DNA, RNA and protein syntheses in HUVE cells*

HUVE cells (2 × 10$^3$ cells) were plated into 96-well Corning cell culture plates precoated with gelatin, and TNP-470 was added to the cultures the next day. Before 4 h from the indicated time, [6-$^3$H]thymidine (74 kBq per well), [5-$^3$H]-uridine (37 kBq per well) or L-[4,5-$^3$H]leucine (74 kBq per well) was added to the wells. In the case of leucine, the medium was replaced with a leucine-poor medium (E-GM – leucine-free RPMI-1640 1:9) containing TNP-470. The plates were incubated for an additional 4 h. Cells were washed with PBS(–) and then trypsinised. Well contents were aspirated onto a fibre filter, washed with distilled water and transferred to scintillation vials using a PHD cell harvester model 290 (Cambridge Technology). The fibre filters in the vials were dried, and a liquid scintilator was added. Radioactivity of the fibre filter was determined.

*Flow cytometric analysis*

For flow cytometric anaylsis, HUVE cells (1 × 10$^5$ cells) were plated into 100 mm cell culture dishes precoated with gelatin. TNP-470 was added to the dishes the next day. After the indicated time, the HUVE cells were washed with PBS(–), trypsinised and centrifuged. The resultant cell pellets were washed with PBS(–) and then fixed with ice-cold 70% ethanol. The fixed HUVE cells were washed, resuspended in PBS(–), treated with RNAse A and stained with propidium iodide. Analysis was performed using an FACScan (Becton Dickinson) interfaced with an HP9000 model 310 computer (Hewlett Packard). Excitation was carried out using the 488 nm line of an air-cooled argon ion laser operating at a continuous output of 15 mW. In order to eliminate the possibility of confusing possible multiplets of G1 cells with ordinary G2 cells, CellFIT software with a doublet discrimination module was used.

**Figure 1** Inhibition of HUVE cell growth by TNP-470. HUVE cells were plated in 24-well plates, and TNP-470 was added to the cultures on the next day. Five days later, the cells were trypsinised and counted with a Coulter counter. The arrow indicates initial cell number. The results are expressed as the mean and standard deviation of four determinations. Invisible error bars are included in the symbols. $^{**}P<0.01$ as compared with the control by using Dunnett-type test.

**Figure 2** Reversibility of the inhibition of HUVE cell growth by TNP-470. HUVE cells were cultured for 2 days with TNP-470 (closed symbols 10 ng ml$^{-1}$ or open symbols 10 µg ml$^{-1}$), washed with fresh medium (day 3) to remove TNP-470, and the culture was continued with (squares) or without (triangles) the inhibitor. The cells were counted with a Coulter counter on the indicated day. The results are expressed as the mean of duplicate determinations. The difference between the mean value and the individual value was within 15% of the mean. Control cells are shown as open circles.

### Results

*Biphasic inhibition of HUVE cell growth by TNP-470*

As shown in Figure 1, TNP-470 inhibited the growth of HUVE cells in a biphasic manner: in the first phase, inhibition of cell growth was not associated with reduction in the cell number below the initial plating number shown with an arrow in Figure 1. The inhibition of HUVE cell growth by TNP-470 in the first phase occurred in a wide range of concentrations (complete inhibition of 0.3–3,000 ng ml$^{-1}$ with IC$_{50}$ of 15 pg ml$^{-1}$) showing a plateau in the dose-response curve. Viability of HUVE cells after incubation at these concentrations was confirmed by the dye exclusion method using methylene blue (data not shown). In the second phase, growth inhibition was observed at concentrations higher than 3,000 ng ml$^{-1}$. The inhibition in the second phase resulted in a reduction in the cell number below the initial plating number. The cells were stained with methylene blue. Furthermore, HUVE cells cultured with TNP-470 at 10 ng ml$^{-1}$ for 2 days, the concentration of the first phase, recovered within 4 days of exchanging the medium for fresh medium without TNP-470 (Figure 2). However, cell growth could not recover after HUVE cells were cultured at 10 µg ml$^{-1}$, the concentration of the second phase. The results indicate that the inhibition in the first phase is cytostatic, and that in the second phase is cytotoxic. In other words, TNP-470 induced cell killing at the concentration in the second phase but not in the first phase.

Duration of cytostatic inhibition of HUVE cell growth was studied by changing cytostatic concentration and incubation time. Complete inhibition of HUVE cell growth continued for 6 days after 2 h incubation at 100 ng ml$^{-1}$, and partial growth inhibition was observed after 16 h incubation at 10 ng ml$^{-1}$ (Figure 3). The cells treated with 100 ng ml$^{-1}$ TNP-470 regrew after longer culture (data not shown).

*Selective inhibition of TNP-470 on thymidine incorporation by HUVE cells*

The effects of TNP-470 on thymidine, uridine and leucine (marker of DNA, RNA and protein syntheses respectively) incorporation by HUVE cells were examined to characterise cell growth inhibition. TNP-470 suppressed [$^3$H]thymidine incorporation at a concentration lower than that required for

**Figure 3** Duration of endothelial cell growth inhibition by TNP-470. HUVE cells were cultured for 2 or 16 h with TNP-470 [10 (▒) or 100 (■) ng ml$^{-1}$], washed and cultured without TNP-470 until day 6. Relative cell number was determined by the MTT method. The results are expressed as the mean and standard deviation of eight determinations. $^{**}P<0.01$ as compared with the control by using Dunnett-type test.

inhibition of either [$^3$H]uridine or [$^3$H]leucine incorporation (Figure 4a). The $IC_{50}$ value for this inhibition is similar to that for inhibition of HUVE cell growth in the cytostatic phase of inhibition. Both [$^3$H]uridine and [$^3$H]leucine incorporation were suppressed at concentrations higher than 3,000 ng ml$^{-1}$, which is similar to those required for the second-phase inhibition of HUVE cell growth. As shown in Figure 4b, the inhibition of thymidine incorporation by TNP-470 was not induced until after 8 h of incubation. Longer incubation, 23 h incubation in Figure 4b, was necessary for the selective inhibition.

**Figure 4** Selective inhibition of DNA synthesis by TNP-470. HUVE cells were cultured for 18 h at varying concentrations of TNP-470 **a**, or at 0.3 ng ml$^{-1}$ of TNP-470 for varying times **b**. Then, [$^3$H]thymidine (O), [$^3$H]uridine (Δ) or [$^3$H]leucine (□) was added, and incubation was continued for an additional 4 h. After incubation, HUVE cells were washed, trypsinised and transferred onto a fibre filter. Radioactivity on the fibre filter was determined. The results are expressed as the mean and standard deviation of four determinations. Invisible error bars are included in the symbols. $^{**}P<0.01$ as compared with the control by using Dunnett-type test.

*Flow cytometric analysis of HUVE cell growth inhibition by TNP-470*

To determine if the inhibitory effects on HUVE cell growth by TNP-470 involved an arrest of the cell growth at a particular phase in the cell cycle, HUVE cells were cultured with TNP-470 at 10 ng ml$^{-1}$ for various periods and then the DNA content of the cells was measured by flow cytometric analysis. The results indicated that an increased proportion of the cells was found in the G0/G1 phase and a decreased proportion of the cells was found in G2/M and S-phases as compared with the controls after 21 h incubation but not after 7 h incubation (Figure 5).

*Growth inhibition of various types of cells by TNP-470*

TNP-470 inhibited the growth of various types of cells besides HUVE cells (Figure 6). All types of cells exhibited sensitivity to TNP-470 more or less with a biphasic inhibition curve but with wide variations in extent of sensitivity. The extent of inhibition of the first phase was variable, although the potency of the inhibition in the second phase was similar. Thus, different types of cells exhibited different $IC_{50}$ values. Growth of rat endothelial cells was inhibited with a similar sensitivity to that of HUVE cells, and these endothelial cells were the most sensitive. On the other hand, the growth inhibition of some types of cells, especially that of tumour cells, was very weak, indicating wide variation in sensitivity to TNP-470 among different cell types.

### Discussion

We previously reported that TNP-470 exhibited potent anti-angiogenic activity in four different assay systems *in vitro* and *in vivo* (Kusaka *et al.*, 1991). In the rat thoracic vein organ culture assay, TNP-470 selectively inhibited capillary-like tube formation, although the mechanism of this selective inhibition is unclear. Therefore, the effect of TNP-470 on HUVE cell growth was examined to clarify its anti-angiogenic activity with special reference to its endothelial cell growth-inhibiting activity.

TNP-470 was found to inhibit the growth of HUVE cells in a biphasic manner: the inhibition in the first phase was reversible, indicating that this inhibition is cytostatic, and the inhibition in the second phase was irreversible and cell number decreased below the plated number, indicating that this inhibition is cytotoxic. Cytostatic inhibition of TNP-470

**Figure 5** Flow cytometric analysis of the effect of TNP-470 on HUVE cells. HUVE cells were cultured for various periods in 10 ng ml$^{-1}$ TNP-470, trypsinised, fixed with 70% ethanol and stained with propidium iodide. Analysis of DNA content was performed with a FACScan flow cytometer.

**Figure 6** Growth inhibition on varoius types of cells by TNP-470. Various cells were cultured with TNP-470 for 4 or 5 days. The cells were counted with a Coulter counter. The results are expressed as the mean of duplicate determinations. The difference between the mean value and the individual value was within 15% of the mean. **a**, HUVE cell (●), rat endothelial cell (△), human embryonic lung fibroblast cell (□), rat smooth muscle cell (○), mouse M5076 reticulum cell sarcoma cell (+). **b**, HUVE cell (●), mouse D14 angiosarcoma cell (○), human leukaemia (HL-60) cell (△), human squamous cell sarcoma (HSC-1) cell (□), Chinese hamster ovary (CHO) cell (+), rat Walker 256 carcinoma cell (×).

on endothelial cell growth was exhibited over a wide range of concentrations from 10 pg $ml^{-1}$ to 3 $\mu g$ $ml^{-1}$. This cytostatic inhibition seems to be important for angiogenesis inhibition by TNP-470 for the following reasons:

1. The concentration for the cytostatic inhibition rather than the cytotoxic inhibition is similar to that for inhibition of capillary-like tube formation, which is a model of angiogenesis *in vitro* (Kusaka *et al.*, 1991).
2. Among fumagillin analogues, those having potent cytostatic inhibitory activity against HUVE cell growth exhibited potent anti-angiogenic activity (Marui *et al.*, 1992), showing a correlation between potencies of cytostatic inhibition and anti-angiogenic action.
3. Serum concentration of TNP-470 was much lower than that for the cytotoxic inhibition after administration of TNP-470 to rats in a preliminary study (manuscript in preparation). Taken together, cytostatic inhibition by TNP-470 seems to be important for its anti-angiogenic and anti-tumour activities.

Cytostatic inhibition of endothelial cell growth by TNP-470 continued for several days even after TNP-470 was removed from the culture medium. The sustained inhibition explains why TNP-470 is effective against tumour growth and metastasis not only upon daily administration but also upon intermittent administration *in vivo*. In fact, TNP-470 was effective even when administered once a week (unpublished data).

Angiogenesis inhibitors have been reported (Bicknell & Harris, 1991; Klagsbrun & D'Amore, 1991). Some of them have inhibitory activity on endothelial cell growth. However, low molecular weight inhibitors of angiogenesis with cytostatic inhibitory activity of endothelial cell growth are scarce. Furthermore, the cytostatic inhibition by TNP-470 is durable after washing out TNP-470 in culture. These characteristics of TNP-470 are beneficial to clinical use. The endothelial cell growth inhibition of the cytostatic type by TNP-470 may be useful because of its lack of toxicity in the treatment of other angiogenic diseases such as arthritis (Peacock *et al.*, 1992).

To characterise the inhibition of endothelial cell growth by TNP-470, biosynthesis of macromolecules (DNA, RNA and protein) in the cells was examined. It was found that TNP-470 selectively suppressed DNA synthesis over a wide range of concentrations. Concentrations for the DNA-specific inhibition were similar to those for the cytostatic inhibition of endothelial cell growth. On the other hand, all of DNA, RNA and protein syntheses of HUVE cells were inhibited at the cytotoxic concentration. The results of flow cytometric analysis confirmed the inhibitory action on DNA synthesis by TNP-470. TNP-470 caused an increased proportion of cells in G0/G1 phases and a decreased proportion in the G2/M and S-phases after more than 21 h of incubation. Selective inhibition of DNA synthesis by TNP-470 may be a result of arrest in the G0/G1 phases and related to the cytostatic inhibition and the low toxicity of this compound.

The mechanism of cell growth inhibition associated with selective suppression of DNA synthesis is not yet clear. TNP-470 arrested HUVE cells in the G0/G1 phases and suppressed DNA synthesis after a lag time of 8 h. Therefore, TNP-470 may function by a mechanism mediated by protein synthesis or protein depletion. In a preliminary experiment, cycloheximide could not rescue growth inhibition by TNP-470, indicating that new protein synthesis is unlikely to be involved in the mechanism of action of TNP-470. The $IC_{50}$ for cytostatic growth inhibition is 15 pg $ml^{-1}$ (37 pM). This low value suggests that TNP-470 may interact in a high-affinity manner with specific molecule(s) that inhibit DNA synthesis either directly or indirectly. Various types of cells showed different sensitivities to TNP-470. Generally, the cell needs growth factor(s) to grow and has signal pathway(s) via receptor(s) on the cell surface for each growth factor(s). So, this different sensitivity may indicate that TNP-470 can discriminate between some unknown responsive sites in growth signal pathways, and the sensitivity or number of the responsive sites may vary depending on the cell type. Furthermore, the relatively low sensitivity of tumour cells to TNP-470 may indicate that the product(s) of proto-oncogene(s) or those of tumour-suppressor gene(s) are involved in the action of TNP-470.

In conclusion, the angiogenesis inhibitor TNP-470 inhibited endothelial cell growth in a biphasic manner. The cytostatic inhibition is accompanied by selective suppression of DNA synthesis. Endothelial cells were the most sensitive to TNP-470. The potent cytostatic inhibition with relative cell specificity on endothelial cell growth by TNP-470 may be related to its anti-angiogenic and anti-tumour activities with relatively few side-effects.

The authors gratefully acknowledge the technical assistance of Ms Eiko Hashimoto.

### References

BICKNELL, R. & HARRIS, A.L. (1991). Novel growth regulatory factors and tumour angiogenesis. *Eur. J. Cancer*, **27**, 781–785.

FOLKMAN, J. (1985). Tumor angiogenesis. *Adv. Cancer Res.*, **43**, 175–203.

FOLKMAN, J. (1990). What is the evidence that tumors are angiogenesis dependent. *J. Natl Cancer Inst.*, **82**, 4–6.

INGBER, D., FUJITA, T., KISHIMOTO, S., SUDO, K., KANAMARU, T., BREM, H. & FOLKMAN, J. (1990). Synthetic analogues of fumagillin that inhibit angiogenesis and suppress tumour growth. *Nature*, **348**, 555–557.

KLAGSBRUN, M. & D'AMORE, P.A. (1991). Regulators of angiogenesis. *Annu. Rev. Physiol.*, **53**, 217–239.

KUSAKA, M., SUDO, K., FUJITA, T., MARUI, S., ITOH, F., INGBER, D. & FOLKMAN, J. (1991). Potent anti-angiogenic action of AGM-1470: comparison to the fumagillin parent. *Biochem. Biophys. Res. Commun.*, **174**, 1070–1076.

MAIONE, T.E. & SHARPE, R. (1990). Development of angiogenesis inhibitors for clinical applications. *Trends Pharmacol. Sci.*, **11**, 457–461.

MARUI, S., ITOH, F., KOZAI, Y., SUDO, K. & KISHIMOTO, S. (1992). Chemical modification of fumagillin. I. 6-O-acyl,6-O-sulfonyl,6-O-alkyl, and 6-O-(N-substituted carbamoyl) fumagillols. *Chem. Pharm. Bull.*, **40**, 96–101.

MOSMAN, T. (1983). Rapid colorimetric assay for cellular growth and survival: application to proliferation and cytotoxic assays. *J. Immunol. Methods*, **65**, 55–63.

PEACOCK, D.J., BANQUERICO, M.L. & BRAHN, E. (1992). Angiogenesis inhibitor suppresses collagen arthritis. *J. Exp. Med.*, **175**, 1135–1138.

WEIDNER, N., FOLKMAN, J., POZZA, F., BEVILACQUA, P., ALLRED, E.N., MOORE, D.H., MELI, S. & GASPARINI, G. (1992). Tumor angiogenesis: a new significant and independent prognostic indicator in early-stage breast carcinoma. *J. Natl Cancer Inst.*, **84**, 1875–1887.